# Point-of-Care Ultrasound Diagnosis of Left-Sided Endocarditis

**DOI:** 10.5811/westjem.2016.2.29921

**Published:** 2016-05-02

**Authors:** Charles W. Bugg, Kristin Berona

**Affiliations:** Keck School of Medicine, LAC+USC Medical Center, Department of Emergency Medicine, Los Angeles, California

A 56-year-old male presented to the emergency department (ED) with fatigue, generalized weakness, cough without hemoptysis or dyspnea, occasional fever, night sweats, dark stools, and weight loss for months. Physical exam revealed a cachectic gentleman with bilateral distal pitting edema. Cardiac and respiratory exams revealed no adventitious sounds. Stool guaiac was negative for blood. Complete blood count revealed mild anemia without leukocytosis, and chest radiograph showed cardiomegaly with left pleural effusions. Initial providers were most concerned for malignancy, although differential included tuberculosis or endocarditis. Given the reassuring exam and chronicity of symptoms, initial disposition plan was discharge with close follow up for malignancy evaluation.

With the consideration of endocarditis, a point-of-care cardiac ultrasound was performed revealing a mobile mass on the aortic valve and an irregular mitral valve (Video). After blood cultures and broad-spectrum antibiotics, the patient was admitted. Transthoracic echocardiogram (TTE), demonstrated vegetations on the mitral and aortic valves. Blood cultures grew enterococcus. The patient underwent mitral and aortic valve replacements without complications and was discharged home.

Infective endocarditis (IE) is an uncommon disease and a challenging diagnosis to make in the ED. Symptoms are nonspecific, murmurs can be difficult to auscultate, and skin manifestations are rare. Though transesophageal echocardiogram is the most sensitive modality for evaluation of suspected IE, it is not available in most EDs. TTE only has sensitivity of 50–70%; however, larger lesions (>10mm) have a sensitivity of 84%.^1^ Vegetations are mobile, irregularly shaped structures usually on the upstream side of valves, with motion irrespective of valve. Differential includes myxomatous process, tumors, thrombi, or imaging artifact.^2^ Point-of-care ultrasound (POCUS) has been reported to diagnose both right- and left-sided IE.^3^,^4^ Though not definitive, POCUS can dramatically change disposition and expedite care in patients for whom emergency physicians are considering IE.

## Supplementary Information

Video Point-of-care ultrasound videos showing aortic and mitral valve vegetations.
